# Role of plant MicroRNA in cross-species regulatory networks of humans

**DOI:** 10.1186/s12918-016-0292-1

**Published:** 2016-08-08

**Authors:** Hao Zhang, Yanpu Li, Yuanning Liu, Haiming Liu, Hongyu Wang, Wen Jin, Yanmei Zhang, Chao Zhang, Dong Xu

**Affiliations:** 1 Symbol Computation and Knowledge Engineering of Ministry of Education, College of Computer Science and Technology, Jilin University, Changchun, China; 2 Department of Computer Science and Christopher S. Bond Life Sciences Center, University of Missouri, Missouri, USA; 3 Institute of Computational Biomedicine, Weill Cornell Medical College, New York, USA

**Keywords:** miRNA, Cross-species regulation, Core module, Functional analysis

## Abstract

**Background:**

It has been found that microRNAs (miRNAs) can function as a regulatory factor across species. For example, food-derived plant miRNAs may pass through the gastrointestinal (GI) tract, enter into the plasma and serum of mammals, and interact with endogenous RNAs to regulate their expression. Although this new type of regulatory mechanism is not well understood, it provides a fresh look at the relationship between food consumption and physiology. To investigate this new type of mechanism, we conducted a systematic computational study to analyze the potential functions of these dietary miRNAs in the human body.

**Results:**

In this paper, we predicted human and plant target genes using RNAhybrid and set some criteria to further filter them. Then we built the cross-species regulatory network according to the filtered targets, extracted central nodes by PageRank algorithm and built core modules. We summarized the functions of these modules to three major categories: ion transport, metabolic process and stress response, and especially some target genes are highly related to ion transport, polysaccharides and the lipid metabolic process. Through functional analysis, we found that human and plants have similar functions such as ion transport and stress response, so our study also indicates the existence of a close link between exogenous plant miRNA targets and digestive/urinary organs.

**Conclusions:**

According to our analysis results, we suggest that the ingestion of these plant miRNAs may have a functional impact on consuming organisms in a cross-kingdom way, and the dietary habit may affect the physiological condition at a genetic level. Our findings may be useful for discovering cross-species regulatory mechanism in further study.

**Electronic supplementary material:**

The online version of this article (doi:10.1186/s12918-016-0292-1) contains supplementary material, which is available to authorized users.

## Background

As a novel mechanism of coevolution, cross-kingdom interactions have recently been discovered in many studies [1–3]. Cell communication can cross species through transmitting signals such as hormones, cytokines and small RNAs (sRNAs) [1]. For instance, *Pseudomonas aeruginosais,* a symbiotic gram-negative bacterium, can produce HSL autoinducers to modulate gene expression in humans [2]. Some of these autoinducers activate epithelial cells to induce generation of neutrophil chemotatic factors and then, these migrated factors will be triggered to produce toxin which are detrimental to the bacteria [2]. Meanwhile, some hosts’ miRNAs can also influence these bacteria. miR-451 and let-7i, which are highly enriched in human HbAS and HbSS erythrocytes, can negatively regulate the growth of the malaria parasite *Plasmodium falciparum* [4]. Certain *Botrytis cinerea* small RNAs (Bc-sRNA), like Bc-siR3.1, can silence *Arabidopsis* and tomato genes to inhibit their hosts’ immunity [3]. It is also reported that milk-derived miRNAs can target infants’ specific transcripts that are involved in cytokines and immunity [5]. Such a cross-species communication also exists in virus-host interaction. Studies have shown that viruses can utilize the hosts’ miRNA machinery to produce their own miRNAs, and further manipulate both virus and host gene expression [6]. Similarly, a cluster of cellular miRNAs, including miR-28, miR-125b, miR-150, miR-223 and miR-382, target the 3′ ends of HIV-1 mRNAs in cultivated resting CD4^+^ T cells to affect HIV-1 latency, and potently inhibit HIV-1 production [7]. Recently, Zhang et al. [8] reported that the exogenous plant miRNA, miR168a, which is enriched in rice, could target the human/mouse low-density lipoprotein receptor adapter protein 1 (LDLRAP1) mRNA, and inhibit LDLRAP1 expression in the liver. Further research [9–11] has also demonstrated that a variety of exogenous plant RNAs can be found in plasma and serum of mammals through ingestion.

Though more and more exogenous plant miRNAs are experimentally detected in the serum and plasma of human and animals, cross-kingdom regulation of human gene by plant miRNAs is not well understood. It is unclear how exogenous miRNAs evade the RNases and phagocytosis, and maintain stable structures and activity in a low-pH environment when passing through the mammalian gastrointestinal tract to reach the target organs. However, all the above evidence suggests that dietary miRNAs might remain active to regulate the ingesters’ specific genes. Although a number of experimental studies have demonstrated the discoveries of cross-species miRNA regulation, the effect of this event and the underlying mechanism are still unclear. Due to the complexity of sRNAs-mRNAs regulations, current experimental studies have some limitations in filling the gaps of those mechanisms. Hence, we carried out a computational study to exploit the functions and effects of plant-mammal cross-kingdom regulations, given the assumption that exogenous miRNAs exist in plasma and serum of mammals. For this purpose, we systematically predicted the targets of the documented cross-species miRNAs, and we conducted function enrichment analysis of the target genes in both humans and plants, by which we could explore the gene sets’ shared functions, such as similar pathways, regulators, or related diseases. In particular, we gathered 25 plant miRNAs, which have been detected in the serum and plasma of human and animals. We collected the entire human mRNAs 3′UTR regions from the UTRdb [12] and predicted the targets of the 25 plant miRNAs from these regions. Interestingly, we found that our predicted cross-species targets might have a close association with the digestive, urinary organs and the daily human metabolic Gene Ontology (GO) process. In order to better understand the functions of the above 25 miRNAs, we also evaluated their targets in plants as a reference. Due to lack of experimental validations on targets and high-quality gene function annotations for common food crops, we used *Arabidopsis thaliana* for plant target prediction and functional analyses. We found the *Arabidopsis* targets share some functional similarity to human targets. Our study might provide some useful hypotheses for discovering the cross-species regulatory mechanism in future research.

## Results

### Target prediction on human and *Arabidopsis* for 25 miRNAs

Due to the limitation of existing studies, it is difficult to exactly define the matching model between plant miRNAs and human targets. Given the notion that most mature miRNAs act as the RNA interference (RNAi) mechanism by binding to certain sites on target mRNAs in both plants and animals [13], we took a basic target prediction approach to retain the maximum potential targets on humans by using RNAHybrid [14] (see Methods). Meanwhile, we used the same protocol to predict the miRNAs’ targets in *Arabidopsis thaliana* for comparative analysis and then, we achieved an initial dataset with nearly 380,000 possible human targets and 5700 *Arabidopsis* targets. After a screening process conducted by selecting filtering parameters regarding targeting attributes, such as the minimum free energy, *p*-value, the length of bulge and loop and so on, finally about 3000 human and 1800 plant targets were selected for further study.

### Predicted target validation on *Arabidopsis*

In order to verify the reliability of our target prediction method, we collected 170 validated *Arabidopsis* targets of these 25 miRNAs from TAIR [15] and PMRD [16]. The results of the *Arabidopsis* target prediction not only showed a high consistency but also shared the exact same binding regions (coding DNA sequence (CDS) or 3′UTR) with validated targets. Out of 170 validate targets, 135 (81.8 %) ranked within the top 50 of each miRNA. More strikingly, 123 out of 170 validated targets ranked in the top ten among the predicted targets. For example, miR156 and miR157 are from the same family, which mainly target SQUAMOSA-promoter binding protein-like (SPL) genes’ coding sequence in *Arabidopsis thaliana*, except for SPL3, 4, 5 located in the 3′UTR [17, 18]. It is reported that members of a plant miRNA gene family often share high sequence similarity and the target site [19]. As shown in Table 1, not only the filtered target genes and the target regions are consistent with this report, but also all validated targets rank on top of predicted targets.

**Table 1:** miR156 target validation

Validated target genes	Accession number	mRNA		Primary targets		Refined CDS targets	Rank	Refined 3′UTR targets	Rank
		CDS	3′UTR	CDS	3′UTR				
SPL2	AT5G43270	Y	Y	Y	N	Y	6	N	
SPL3	AT2G33810	Y	Y	N	Y	N	-	Y	1
SPL4	AT1G53160	Y	Y	N	Y	N	-	Y	2
SPL5	AT3G15270	Y	Y	N	Y	N	-	Y	3
SPL6	AT1G69170	Y	Y	Y	N	Y	5	N	
SPL9	AT2G42200	Y	Y	Y	N	Y	2	N	
SPL10	AT1G27370	Y	Y	Y	N	Y	4	N	
SPL11	AT1G27360	Y	Y	Y	N	Y	3	N	
SPL13-1	AT5G50570	Y	Y	Y	N	Y	7	N	
SPL13	AT5G50670	Y	Y	Y	N	Y	8	N	
SPL15	AT3G57920	Y	Y	Y	N	Y	1	N	

We examined whether the validated genes exist in the original mRNA dataset, the primary predicted target set by RNAHybrid, and the refined targets after screening. We also listed the rank of each validated target in our refined target set, which is sorted by minimum free energy (MFE) and *p*-value.

### Reconstruction of cross-species regulatory network

**Fig. 1:**
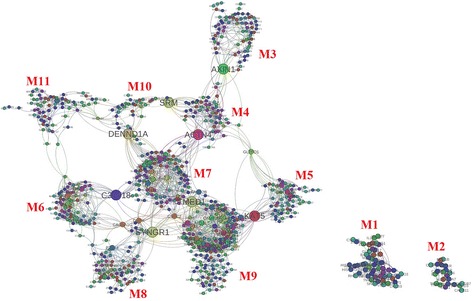
Integrated network of human target genes. The nodes with bigger size represent the bridge genes (AXIN1, SRM, DENND1A, ACTN4, C3orf18, TMED1, KAT5, and SYNGR1) in the reconstructed network

We extracted experimentally verified interactions and signaling networks/pathways for our 531 filtered targets (see Additional file 1) using GeneMania [20], and then created a primary regulatory/interaction network containing 782 genes and 2444 interactions in total (Fig. 1).

The PageRank algorithm was adopted to assign weights to the 531 nodes and thereby measure their importance. The results were convergent after 25 iterations (Additional file 2: Figure S1), when the perturbation of the weights could be controlled within 0.005. Then, we selected the top 15 nodes as the bridge genes to reconstruct the initial network by extracting modules. We extracted 11 modules by taking the bridge genes as the central nodes to decompose the whole network, which can be regarded as a critical link in the biological network.

### Three functional categories of modules

**Fig. 2:**
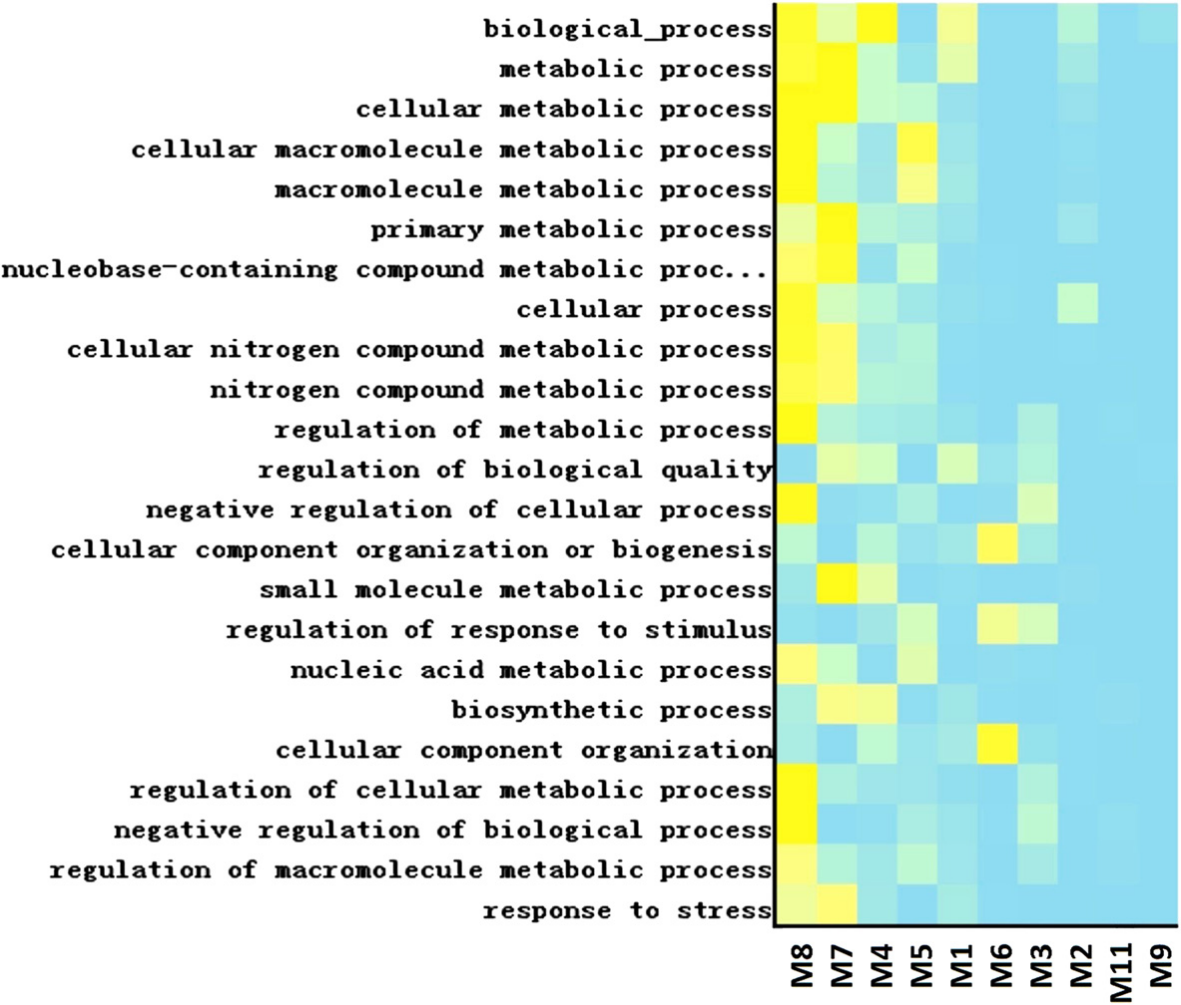
Heatmap of common biological process functions in different modules

In our study, functional enrichment analysis was applied to the above modules individually using Mosaic and NOA Cytoscape apps [21, 22] and DAVID [23], and we found seven modules highly enriched in certain functions. These modules could be classified into three functional categories: 1) Transport: ion transport and homeostasis process, 2) Other metabolic process: macromolecule biosynthetic and metabolic process and 3) Stress response: immune and stress response (Table 2). Major commonly enriched biological processes functions of the main modules are shown in Fig. 2 (M9, M10 and M11 are excluded, since none of the listed functions are enriched in these modules). The ion transportation and homeostasis process includes chemical, di- and tri-valent inorganic compounds (such as boron and sulfur), which are often enriched in fruits, leafy vegetables, and cereal. Metal cations such as iron, calcium, and sodium are also present. All of these compounds are closely linked with dietary habits. Meanwhile, we found a tight connection between the predicted human tissue targets as they relate to fatty acids, amino acids, alcohol, glycerolipids, cellular polysaccharide, and oxoacid metabolic processes. For the response process, one is the metal ion, inorganic ion and the nutrient substance related response, and the other is a type of defense response to the external stimulus, such as virus, bacterium and wounding. For the immune process, the identified modules show positive regulation of leukocyte and lymphocyte activation with response to the immune effector process as well as inflammation. M6 is related to vesicle targeting and transportation as well, which has been regarded as a carrier of the uptake miRNAs in the human body.

**Table 2:** Parts of the functions of selected modules

Function	Specific process	BP accession number	Module
Transport	iron ion homeostasis	GO:0055072	M1, M3
	calcium ion transport	GO:0006816	M6
	vesicle-mediated transport	GO:0016192	M8
	vesicle targeting, to, from or within Golgi	GO:0048199	M8
	vesicle targeting	GO:0006903	M8
	regulation of sodium ion transport	GO:0002028	M8
Other metabolic process	glycerolipid biosynthetic process	GO:0045017	M4
	biogenic amine metabolic process	GO:0006576	M4
	alcohol metabolic process	GO:0006066	M4
	cellular polysaccharide biosynthetic process	GO:0033692	M4
	polyamine metabolic process	GO:0006595	M5
	fatty acid biosynthetic process	GO:0006633	M7
	fatty acid metabolic process	GO:0006631	M7
Stress response	innate immune response	GO:0045087	M1, M2
	inflammatory response	GO:0006954	M1, M2
	response to molecule of bacterial origin	GO:0002237	M2
	response to wounding	GO:0009611	M2
	response to chemical stimulus	GO:0042221	M2,M7
	leukocyte chemotaxis involved in inflammatory response	GO:0002232	M2
	response to ethanol	GO:0045471	M4
	response to heat	GO:0009408	M7
	response to temperature stimulus	GO:0009266	M7, M8

### Functional analysis of tissue-specific predicted human targets

The range of the protein expression level varies greatly in different tissues; thus, we took tissue specificity as a basis to measure our targets, which could largely reduce the false positive rate of the predicted results. To investigate the relationship between our cross-species targets and the eight tissues (brain, heart, kidney, liver, lung, spleen, stomach, and small intestine), we categorized our target genes into three levels, the ubiquitous level (house keeping genes, HKG), for which genes would express in most of the tissues; highly expressed level, meaning these genes would have a high expression level in several but not all tissues; and the tissue-specific level, which can be specifically expressed within one certain tissue. To characterize the set of three-level expressed genes that we had identified, we conducted functional enrichment analysis, and for the third level we collected verified specific gene sets of the eight tissues from TisGed [24] as the background. The protein products of the first level expressed genes were more likely to be involved in the actin filament-based process, ion transport and signaling (calcium, sulfate, and organic anion), metabolism process such as those found in macromolecule (polysaccharide, carbohydrate, alcohol, and ATP) biosynthetic and metabolic processes, general cell morphogenesis and apoptosis processes, and vesicles localization and targeting.

**Fig. 3:**
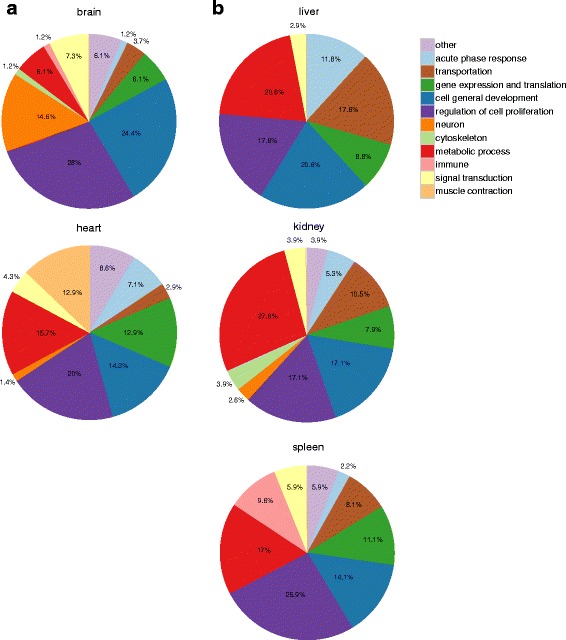
Function distribution of the high level targets among five tissues. Pie graphs show fractions of cellular Biological Processes (BP) derived from genes belonging to the second expression level in six human tissues. Names of BP categories are shown at the right. **a** The BP terms of brain and heart are more likely to be related with regulation of cell proliferation. **b** For liver, kidney and spleen, functions related to transportation, the metabolic processes and immune-related processes are highly enriched

As shown in Fig. 3, genes with a specific tissue expression level in the brain and heart were more likely to be connected with general cell development as well as the neurological system related process, and cardiac muscle tissue morphogenesis. While the second and third level targets in kidney, liver, and spleen were often involved in specialized biological processes, like metal ion transportation, homeostasis and response in kidney, immune system process and wounding response in spleen, alcohol and macromolecule metabolic processes, and insulin signaling pathway, as well as the catabolic process in the liver, which suggests a high enrichment both in the digestive and urinary system, and also indicates a close link between our targets and these systems.

### Functional similarity: ion transport and stress response between *Arabidopsis* and human

As *Arabidopsis* and human are two very different species, there is no established method to compare gene functions of them and no single cross-species platform or algorithm can perform the pathway analysis on both *Arabidopsis* and human. The “functions” mentioned in this paper refers protein functions annotated in Gene Ontology. According to our enrichment analysis results (Additional files 3, 4 and 5), ion transport and stress response exist in most modules from both *Arabidopsis* and human. In plants, there are two ways to acquire iron ions: based on iron reduction and iron chelation. *Arabidopsis* activates the reduction-based strategy of Fe uptake upon Fe deficiency, and this process is partly induced by an acidification of the root hair zone through the extrusion of protons to solubilize Fe chelates; hence, this iron ion transport pathway can regulate the growth of root. Also, some transporters are expressed in shoots and seedlings. Fe distributes in three main organelles in cell: vacuole, chloroplast and mitochondria, and the iron ion transport process can influence mitochondrial respiration and chloroplast photosynthesis [25]. In *Arabidopsis*, the Fe uptake pathway appears to be regulated at the transcriptional and post-transcriptional level. At the transcriptional level, the transcription of IRT1genes is strongly up-regulated, IRT1 is the founding member of the large ZIP family [26], and our predicted target genes involved in metal ion transport include ZIP family gene. Two genes AtNRAMP3 and AtNRAMP4 are also involved in this pathway [27]. In human, extracellular Fe^3+^ is reduced to the more soluble Fe^2+^ by reductases embedded in the cell’s plasma membrane. The Fe^2+^ generated then becomes the substrate for two different uptake systems, a high-affinity system expressed in iron-limited cells and a low-affinity system active in iron-replete cells. The high-affinity system is just the same as in *Arabidopsis*. In the human iron transport pathway, some genes involved may influence organs such as livers and so on [25]. One mainly gene is NRAMP2 [28], and AtNRAMPs show homology to the NRAMP family [27]. We find a predicted target gene FER involved in human iron transport is also involved in the plant iron transport pathway according to our prediction result.

Plants and animals both suffer from external stimuli and pressure all the time, which helps them to constantly adapt to environmental stressors by improving the ability to respond to threats during evolution [29]. By applying the same module extraction and functional analysis method to our *Arabidopsis* targets (see Additional file 3), we found two aspects of stress response: 1) response to stimulus, (e.g., probable histone H2A variant 3), and 2) the more specific defense response as exhibited by the protein MOS2 resisting bacterium. The putative DNA repair protein RAD23-1 responds to DNA damage with stimulus reactions. The other defense response could be seen in the pyruvate dehydrogenase E1 component subunit alpha-2 and mitochondrial protein, which helps to repel salt stress and osmotic stress. Meanwhile, we found that predicted human targets could play a prominent role in both the stress response and immune process related to 106 relevant GO processes, of which 72 pieces are external response processes, which shows a high similarity to that of plants. Similarities can be seen in plant responses to metal ions, inorganic salt and unhealthy nutrient levels as well as starvation. All of these processes can be compared to biotic and abiotic stresses whose main factors are water and nutrients. We also found a high rate of external stimulus response in plants including cellular responses to molecules of bacterial origin, responses to lipopolysaccharides (LPSs), which are the main component of the gram-negative bacteria that can protect them from chemical attack. Gram-negative bacteria also respond positively to wounding and inflammation. Module M3/AXIN1 (Axis inhibition protein 1), which is associated with 26 genes, which involve myeloid leukocyte mediated immunity, activation of blood coagulation via a clotting cascade, glucagon secretion, and immune response-inhibiting signal transduction, which forms the immune system process.

## Discussion

*Arabidopsis* and human are very different species, and it is hard to define “functional similarity” between them according to current computational tools and databases. We used three most comprehensive function/pathway databases, GO, KEGG and BioCyc to perform function enrichment analysis. We understand that most GO terms present very generic functions, but so far GO is the most comprehensive functional annotation database for both *Arabidopsis* and human. In KEGG database, the number of pathways for *Arabidopsis* and human are 133 and 299, and those pathways only covered 4818 and 6997 genes, respectively. Due to the unbalance of annotations between *Arabidopsis* and human, we cannot compare involved pathways between them. But according to our enrichment analysis results and literature search, we still find some interesting results, which infer that the pathways between *Arabidopsis* and human are not only common at the generic level, but also have a similar mechanism in the specific process.

According to our enrichment analysis results, one of the enriched functions is ion transport (GO: 0006811) in *Arabidopsis* and some GO terms about metal ion transport also exist in human, such as iron ion transport (GO: 0006826). In plants, iron is potentially highly toxic to cells, and hence, iron homeostasis needs to be tightly regulated. Similarly, dietary Fe deficiency affects many human beings on earth, leading to asthenia, increased sensitivity to diseases, and even death. We predict human targets using *Arabidopsis* miRNAs in our research, and then we find some predicted targets involved in iron transport process both in human and plant, such as FER. As we use *Arabidopsis* miRNAs to predict human targets, we find a predicted target gene FER involved in human iron transport, and this gene is also involved in the plant iron transport pathway.

Plants have to endure various stresses such as drought, salt, low temperature, etc. These adverse abiotic and biotic environmental factors force plants to develop their stress response mechanisms through cell signaling, genetic regulatory adaptation, and other defense responses. The human body can also respond to stresses and external stimuli, a state of perceived threat to homeostasis, by activating the immune system. In our research, we found that potential cross-species targeted by the exogenous miRNAs are highly related to the immune system and stress response process in the human body, such as the response to chemical stimulus and the defense response to bacterium. These responses are in the same category of the genes targeted by miRNAs in native plants. In other words, the exogenous miRNAs in humans may mimic the indigenous miRNAs in plants in terms of biological functions. So far, there exists no connection between these two response mechanisms. It is reported the stress response is generally transient because its accompanying effects can be harmful in the long term. However, if these exogenous miRNAs exist in the human chronically from the daily food consumption, they may have lasting evolutionary effects on a human population with similar food sources.

The cross-species regulatory mechanism was not proposed until 2012, and significant discussion (both pro and con) continues on the validity of this proposal. To the best of our knowledge—up to now—no validated method has been developed to predict miRNA targets across species for the exact binding site. Given the common mechanism of mature miRNAs:RNAi binding, we only applied basic target binding principles in our primary prediction step. Meanwhile, we used the same method to predict the *Arabidopsis* targets as a test benchmark. The significant consistency between our predicted plant targets and the validated ones for this benchmark strongly supports the effectiveness of our method and parameters. Our method tuned using *Arabidopsis* target validation gives a remarkable reduction of the noisy points in our data (Additional file 6: Figure S2) and, hence, provides a valid guidance to explore the new mechanism of cross-species miRNA targets.

## Conclusions

In conclusion, we present a novel computational method to study the cross-species regulation between human genes and plant miRNAs. According to the target genes in both human and *Arabidopsis* we predicted for the same 25 miRNAs and the cross-species regulatory network we built, we summarized the functions to several major categories. From these functions, we found that there are some similar functions between human and plant target genes, such as ion transport and stress response. These findings may provide a hint of transcriptional regulatory interactions between human and plants through miRNAs. And it might point a new direction to understand the biological processes in human body through the cross-species regulatory mechanism in the future.

## Methods

### Plant species selection and original datasets

Related studies have detected miRNAs of *Oryza sativa* and *Glycine max* in animal serum, but we chose miRNAs in *Arabidopsis thaliana* for our analyses for the following reasons. A miRNA family often shares similar nucleotide sequences among different plant species. *Arabidopsis* is a model organism [30–32], and compared with other plants, it has more data and annotations. A total of 25 Arabidopsis miRNA datasets and 35,173 mRNAs were retrieved from the *Arabidopsis* Information Resource (TAIR) [15] and PMRD [16], and 34,619 human 3′UTR were from UTRdb [12]. We observed that most of these 25 miRNAs widely exist in many plants with high expression levels (Table 3), which means that human can easily intake significant quantity from common plant foods.

**Table 3:** Expression levels of exogenous plant miRNAs in common plant foods

Exogenous miRNAs	*Brassica napus*	*Glycine max*	*Oryza sativa*	*Sorghum bicolor*	*Solanum lycopersicum*	*Vitis vinifera*	Zea mays
miR156a	√	√	2.72E+05	3.09E+02	2.98E+03	2.08E+03	1.24E+03
miR156g	√	√	2.72E+05	1.34E+03	NA	1.78E+03	572
miR157a	NA	NA	NA	NA	NA	NA	NA
miR157d	NA	NA	NA	NA	NA	NA	NA
miR159a	NA	√	5.91E+03	966	NA	8.02	6.41E+03
miR160a	√	√	1.32E+03	3.1	75.6	32.7	12.7
miR162a	√	√	NA	NA	NA	1.04E+04	NA
miR164a	√	√	3.99E+03	81.3	1.81E+04	301	88
miR164c	√	√	3.41E+03	22.9	NA	1.24E+04	51.1
miR165a	NA	NA	NA	NA	NA	NA	NA
miR166a	√	√	4.03E+04	643	2.92E+05	2.25E+04	8.00E+03
miR167a	√	√	1.51E+04	1.66E+03	1.42E+04	1.34E+03	1.29E+03
miR167d	√	√	1.57E+04	3.02E+03	NA	548	180
miR168a	√	√	3.58E+05	NA	√	NA	1.47E+05
miR169a	√	√	8.17E+03	387	47.4	23	318
miR169b	√	√	9.83E+03	120	NA	4.59	58.8
miR169h	√	NA	NA	NA	NA	104	NA
miR171a	√	√	3.24E+03	12.3	206	212	√
miR171c	√	√	2.71E+03	NA	85.3	536	17
miR172a	√	√	1.72E+04	6.44E+03	1.27E+04	8.6	73.3
miR172c	√	√	1.48E+04	91.3	NA	3.21E+03	5.28E+03
miR390a	√	√	NA	NA	2.83E+03	NA	236
miR394a	√	√	NA	155	NA	145	56.9
miR397a	√	√	827	NA	NA	1.25E+03	NA
miR408	NA	NA	790	8.50E+03	NA	1.69E+03	NA

Here the numbers represent the reads per million; “√” means that the miRNA is found in the species but its expression level is unavailable; ‘NA’ means that there is no evident to support this miRNA

### Target prediction by RNAhybrid

RNAhybrid [14] was chosen as the target prediction tool with criteria of “seed region rules” (perfect nucleotide match at the core sequence that encompassed the first two to eight bases of the mature miRNA); the free energy of the hybrid was expected to be within the range of the authentic miRNA-target pairs, typically lower than −25 kcal/mol. For *Arabidopsis* target prediction, we also applied an additional restriction on the length of loop and bulge within 5 and 9 nt, respectively.

### Plant target validation

To test the reliability of our targets, we designed a three-level validation method. First, we checked whether this validated target existed in our input mRNA dataset; second, we checked the primarily predicted targets to ensure the effectiveness of our predicting method; third, we checked whether it was excluded after the screening process to evaluate the performance of our screening parameters. After the three-level test, we ranked the target by its *p*-value first, then the MFE, and set a threshold to include most of the validated targets.

### Node weight assignment

According to the connectivity among all the N nodes in the network, we used the PageRank [33] algorithm to evaluate each node by constructing an N*N matrix A. As a famous algorithm used by Google, PageRank has proved to be a good measurement of the importance of website pages. If there is a connection between two nodes, we set a value of one to the corresponding position of matrix with all the rest set at zero. Through this process, we recorded all edges in A. We also used a 1*N-dimensional matrix B_0_ to store each node’s original weight, which is designed equally as one. Then, we multiplied the matrix A and B_0_ to obtain B iteratively. After about ten iterations, we obtained a converged matrix B as the final weight of each node.

### Core module selection

According to the relationship between any two nodes, we took the entire network as an equivalent of an undirected, connected graph G. The core module selection process is based on the bridge gene v, to extract sub-graph G’ from G. The bridge genes rank as nodes with the highest connectivity; thus, we derived the modules by simply deleting these bridges from G, so the G graph was changed into 11 separate highly connected components.

### Functional analysis on network and modules

Two Cytoscape apps, Mosaic [21] and NOA [22], were used to analyze the functional similarity among the different human target sub-networks. The GO biological process terms were assigned to each gene in the module using Mosaic, and then all sub-networks were analyzed together under the “Batch mode” of NOA with the complete network as the background. In order to control the type I error rate of multiple hypothesis testing, the Benjanmini & Hochberg method was employed to adjust *P-values*, so that GO terms were considered as statistically significant in overrepresented functions with adjusted-*P* <0.1. The functional heatmap (Fig. 2) was generated by NOA.

Since NOA app does not have the annotation for *Arabidopsis*, in order to compare the functional similarities of modules between *Arabidopsis* and human, we performed the functional enrichment analysis on each detected modules/sub-networks for both *Arabidopsis* (Additional file 3) and human (Additional file 4) with DAVID [23]. The GO biological process terms were considered to be statistically significant with DAVID’s default threshold *p*-value <0.1. Then a web-based tool CateGOrizer [34] was used for simplifying enriched functions and grouping them into more generic GO categories for (Additional file 5). The functional similarity between *Arabidopsis* and human was measured by calculating the overlaps of two generic GO categories obtained from the CateGOrizer.

## Abbreviations

AXIN1, Axis inhibition protein 1; Bc-sRNA, *Botrytis cinerea* small RNA; CDS, coding DNA sequence; GI, gastrointestinal; GO, gene ontology; HKG, house keeping genes; LDLRAP1, low-density lipoprotein receptor adapter protein 1; LPSs, lipopolysaccharides; MFE, minimum free energy; miRNAs, microRNAs; RNAi, RNA interference; SPL, SQUAMOSA-promoter binding protein-like; sRNAs, small RNAs; TAIR, the *Arabidopsis* Information Resource

## Additional files

Additional file 1: Supplementary Information. It contains the input of network construction (Data S1) and verified tissue-specific gene selection (Data S2). (PDF 132 kb) Additional file 2: Figure S1. Node weight assignment. (a) Node weight distribution of all 531 nodes. (b) Weight changes during iterations. The change was reduced to within 0.003 after 15 iterations. (PDF 8264 kb) Additional file 3: The technical details and p-values to describe the enrichment of GO terms for *Arabidopsis*. (XLS 109 kb) Additional file 4: The technical details and p-values to describe the enrichment of GO terms for human. (XLS 96 kb) Additional file 5: The result of grouping generic GO categories for *Arabidopsis* and human individually by CateGOrizer. (XLS 23 kb) Additional file 6: Figure S2. Comparison between plant and human target distributions after the filtering process. (a) Original *Arabidopsis* targets. (b) *Arabidopsis* targets after screening. (c) Original Human targets. (d) Human targets after screening. There is a remarkable reduction of the noisy points between (a) and (b), and between (c) and (d), which strongly supports the effectiveness of our method and parameters, and provides a valid guide that can help explore the mechanism of cross-species miRNA targets. (PDF 8744 kb)
