# Supplementary material for: Role of plant MicroRNA in cross-species regulatory networks of humans
Source: BMC Syst Biol. 2016 Aug 8;10:60. doi: 10.1186/s12918-016-0292-1 (PMC4977847; doi:10.1186/s12918-016-0292-1)
Supplement: Additional file 1: — Supplementary Information. It contains the input of network construction (Data S1) and verified tissue-specific gene selection (Data S2). (PDF 132 kb) [file 12918_2016_292_MOESM1_ESM.pdf]

Role of Plant MicroRNA in Cross-species Regulatory Networks of Humans

Hao Zhang[1,2], Yanpu Li[1], Yuanning Liu[1,2], Haiming Liu[1], Hongyu Wang[1], Wen Jin[1], Yanmei Zhang[1], Chao Zhang[3], and Dong Xu[1,2,*]

Supplementary Information, Data S1

Input of network construction

After the target validation process, we set a threshold of 50 genes, meaning that for each miRNA, we only select top 50 targets. For all the 25 miRNAs, we derived 531 targets after removing the duplication.

A4GALT	CDC42EP2	GPC1	MEST	S100A2	VPS33B
AATF	CDCA5	GPNMB	MFI2	S100A9	VPS53
ABCB9	CDK20	GPR115	MFSD7	SAMD14	WDR11
ABI3	CDT1	GPR52	MIDN	SAP30BP	WDR49
SPPL2B	CECR5	GPRC5B	MMP25	SASH3	WDR83
AC007952.1	CEND1	GPRIN1	MORC2	SCNN1G	WFDC10B
ACP2	CHRM1	GPT	MRPL24	SCUBE1	WFDC2
ACSF2	CINP	GRASP	MRPS2	SDF4	WIPI1
ACTN4	CLDN7	GRIPAP1	MSI1	SDHAF2	WNT10A
ADAM18	CLEC11A	GRN	MTFP1	SEMA4C	WWOX
ADRA2C	CLPTM1	GSDMC	MXD3	SEMA4G	XDH
AKAP10	CLPTM1L	GTF3C5	MXRA8	SEMA6B	XPO6
AKR7A2	CNTN1	GUCY2F	MYO1A	SEPX1	XRCC3
AKT1	COQ4	GYS1	MYOC	SERPINA3	ZBTB20
ALKBH4	CPN2	H1FX	MZF1	SERPINB8	ZC3H18
ALPPL2	CPZ	HES6	NAT6	SERPING1	ZDHHC1
AMIGO3	CREB3	HGF	NDEL1	SETD6	ZDHHC11B
ANKRD34A	CREG1	HIST1H2BO	NDUFAF1	SF1	ZFP62
AP1B1	CRIP1	HIST2H3A	NEFM	SF3B5	ZG16
AP1M2	CRTC2	HIST2H3C	NEU4	SH2D3C	ZMYND11
AP3S1	CSDC2	HLA-DMA	NF2	SIDT2	ZNF221
ARFGAP1	CSF2RB	HMHA1	NGFRAP1	SIRT3	ZNF259
ARHGAP6	CST9L	HOMER3	NKAIN4	SIRT6	ZNF362
ARHGDIG	CTSZ	HOXB1	NR0B2	SIX2	ZNF497
ARHGEF5	CTXN1	HOXB5	NR5A1	SIX6	ZNF584
ARMCX6	CXorf48	HOXD4	NT5M	SKP1	ZNF595
ARSD	CYP2A7	HOXD9	NUDT18	SLC25A10	ZNF628
ASCL2	D2HGDH	HSF2BP	NUDT2	SLC25A22	ZNF645
ASH2L	DBH	HSPA12B	OLFM2	SLC25A29	ZNF771

ASPRV1	DBNDD2	HSPB2	OLIG2	SLC26A1	ZNF789
ATAD3A	DBNL	HSPB8	OR2AG1	SLC29A2	ZNRF4
ATOX1	DDT	HTR3E	OXER1	SLC29A4	
ATP10A	DDX31	HTR4	P2RX4	SLC2A4RG	
ATP13A1	DDX47	IDH3G	P2RY11	SLC2A5	
ATP13A2	DDX54	IFT74	PACSIN3	SLC32A1	
ATP4A	DEDD	IGFBP7	PAICS	SLC38A10	
ATP5D	DENND1A	IGSF22	PAK4	SLC4A11	
ATP5SL	DFNA5	IL18RAP	PAQR6	SLCO5A1	
ATP6V0A4	DIAPH1	IL20RB	PAQR7	SMG6	
ATPAF2	DISC1	ILVBL	PBX4	SMTNL2	
ATPIF1	DMPK	INF2	PDE1C	SNAI1	
AXIN1	DNAI2	INSC	PDZD7	SNAP47	
B2M	DNAJB13	INSM2	PEA15	SND1	
B3GALT6	DNMT1	IRF5	PEAR1	SNRPB	
B4GALNT4	DOLPP1	IRX1	PEMT	SOX14	
BARX1	DTNA	ISL2	PHYHD1	SPATA2L	
BBS1	DUSP13	ISOC2	PID1	SPON2	
BCL7B	DUSP7	ITM2C	PIN1	SPTBN4	
BCS1L	E4F1	JAKMIP2	PITX3	SPTLC1	
BEST4	EEF1A2	KAT5	PKP3	SRM	
BLK	EFNA3	KCNJ4	PLA2G4C	SRRM3	
BRCC3	EFNB1	KCNK12	PLAT	SRRM5	
BUB3	EIF2C2	KCNN2	PLIN1	ST6GALNAC5	
C10orf10	ENO1	KIAA0100	PLK1	STK25	
C11orf21	EPHA8	KIAA0913	POU4F3	STK32C	
C11orf49	ERICH1	KIF26A	PPP1R9B	STOX2	
C11orf73	ERN1	KIF2B	PPP2R5D	SURF6	
C12orf74	ERN2	KIFAP3	PPYR1	SUSD1	
C15orf24	ESPNL	KIR2DL1	PQLC1	SYNGR1	
C15orf44	ESRRA	KIR2DL5B	PRAMEF10	SYTL1	
C17orf105	ETV3	KIRREL3	PRDM2	TAB1	
C17orf48	EXOC6	KLF15	PRELID2	TBC1D10B	
C17orf62	EXT2	KLK3	PRKAR1B	TCAP	
C17orf70	FADS6	KNG1	PRKCD	TCF21	
C19orf22	FAH	KRT34	PRPH	TCTN2	
C19orf25	FAHD2A	KRTAP13-1	PRPS1	TDRKH	
C19orf43	FAHD2B	KRTAP9-3	PRR18	TECPR1	
C1orf109	FAM103A1	KRTAP9-4	PRSS21	TGM7	
C1QL2	FAM109B	KRTAP9-8	PRSS35	TMCO5A	
C20orf132	FAM123A	LAMP1	PRTFDC1	TMED1	
C20orf20	FAM204A	LBP	PTP4A3	TMEM114	
C20orf24	FAM205A	LBX2	PTPN5	TMEM129	
C2CD2L	FAM206A	LCE1B	PUSL1	TMEM159	

C3orf18	FAM22A	LCE1C	PXK	TMEM175	
C3orf20	FAM22F	LCN1	PYY	TMEM180	
C4orf44	FAM40A	LCP1	QRICH1	TMEM203	
C6orf108	FBXL19	LECT2	RAB37	TMEM79	
C6orf201	FBXO25	LGALS7	RABEP2	TMPRSS3	
C6orf26	FDXR	LGALS7B	RABGAP1	TNFRSF17	
C6orf47	FGFR4	LGI4	RAMP1	TP73	
C7orf10	FOLR2	LIMS2	RANGAP1	TPI1	
C7orf34	FOXM1	LINC00482	RARG	TRADD	
C7orf68	FTH1	LPAR3	RBM19	TRAF2	
C9orf24	FZR1	LRRC41	RBP7	TRAPPC2L	
CABIN1	GABRE	LRRC49	RCC1	TRIM11	
CACNA1G	GALNS	LRRC6	RETN	TRIM28	
CACNG7	GATA6	LY6D	REXO4	TRIM54	
CAMK2N2	GBAS	MAFF	RGS10	TRMU	
CAPN10	GCAT	MAGIX	RHBDD3	TRO	
CAPN3	GDF15	MAP3K11	RHOF	TST	
CAPZB	GGA1	MAPK13	RNF135	TUBB3	
CARS	GIPR	MAPK4	ROGDI	TYMS	
CCDC107	GJB1	MAPK8IP2	ROR1	U2AF2	
CCDC64B	GJB3	MAPRE3	RP1L1	UBL4B	
CCDC77	GJB4	MARCKSL1	RPL13	ULK4	
CCDC88C	GLT1D1	MATN4	RPS6KA4	UMODL1	
CCM2	GLTPD1	MBD2	RPS9	UNC5B	
CCNE1	GMPR	MBD3	RPUSD1	UPK3B	
CCS	GNLY	MC5R	RSPO1	UTP18	
CCT7	GORASP1	MCAT	RTN4RL1	VENTX	

Supplementary Information, Data S2

Verified tissue-specific gene selection

We collected the validated specific genes of eight tissues from TiSGed [23]. For this collection, we set certain domains of the parameters (SPM and DPM) as designed by TiSGed.

Gene Level	SPM	DPM
**Ubiquitous**	> 0.2	> 0.2
**High**	< 0.7	< 0.7
**Specific**	< 0.9	< 0.9

SPM and DPM both ranged from 0 to 1. SPM is a parameter indicating tissue-specific genes when it is close to 1, whereas DPM is especially for ubiquitous genes when approaching 0. By using both SPM and DPM values, we gain confidence in filtering out the genes at each level.

Figure Legends

Supplementary Information, Figure S1

Figure S1. Node weight assignment. (a) Node weight distribution of all 531 nodes. (b) Weight changes during iterations. The change was reduced to within 0.003 after 15 iterations.

Supplementary Information, Figure S2

Figure S2. Comparison between plant and human target distributions after the filtering process. (a) Original Arabidopsis targets. (b) Arabidopsis targets after screening. (c) Original Human targets. (d) Human targets after screening. There is a remarkable reduction of the noisy points between (a) and (b), and between (c) and (d), which strongly supports the effectiveness of our method and parameters, and provides a valid guide that can help explore the mechanism of cross-species miRNA targets.

Supplementary Information

Supplementary Information File 1 contains the input of network construction, verified tissue-specific gene selection, node weight assignment and comparison between plant and human target distributions after the filtering process.

Supplementary Information File 2 and File 3 are the technical details and p-values to describe the enrichment of GO terms for Arabidopsis and human, respectively.

Supplementary Information File 4 is the result of grouping generic GO categories for human and Arabidopsis individually by CateGOrizer.
