# Supplementary figures and images for: Role of plant MicroRNA in cross-species regulatory networks of humans
Source: BMC Syst Biol. 2016 Aug 8;10:60. doi: 10.1186/s12918-016-0292-1 (PMC4977847; doi:10.1186/s12918-016-0292-1)

A)

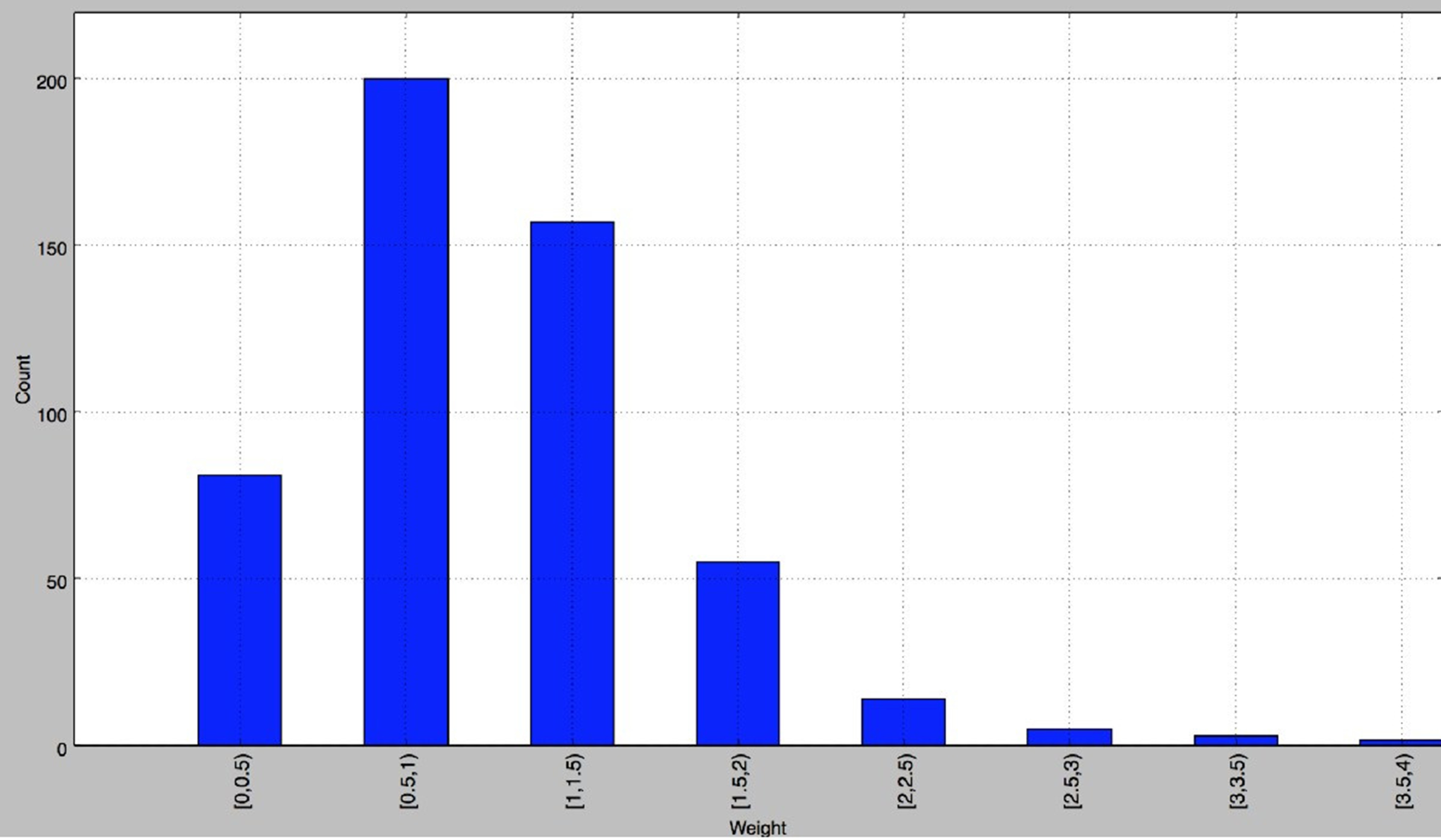

B)

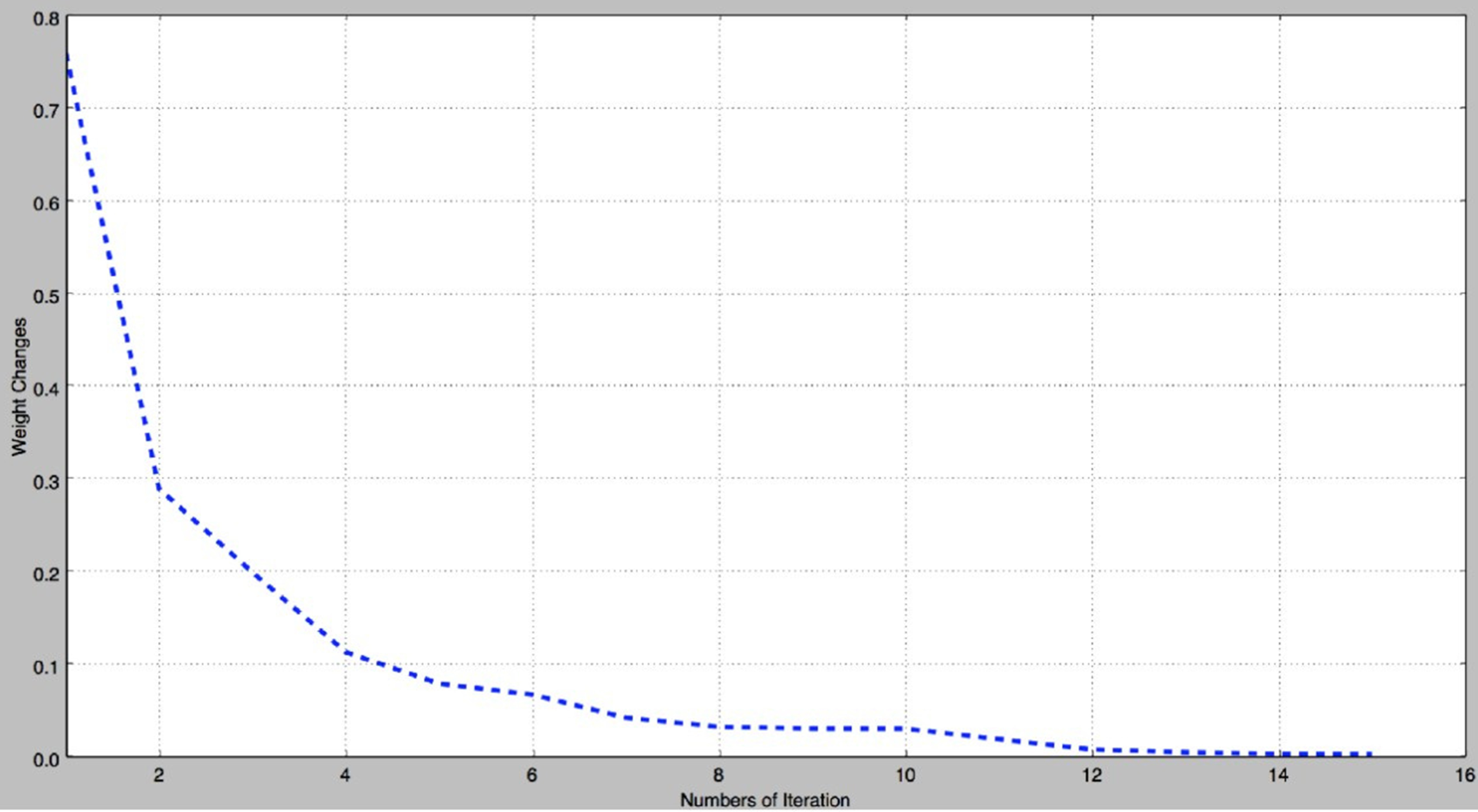

Supplement: Additional file 2: Figure S1. — Node weight assignment. (a) Node weight distribution of all 531 nodes. (b) Weight changes during iterations. The change was reduced to within 0.003 after 15 iterations. (PDF 8264 kb) [file 12918_2016_292_MOESM2_ESM.pdf]

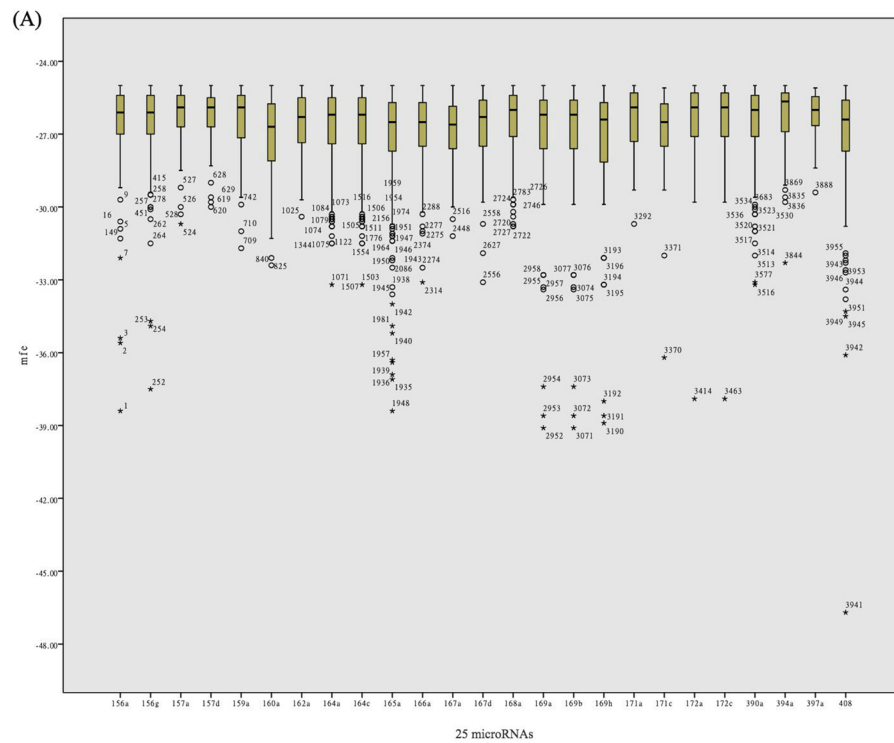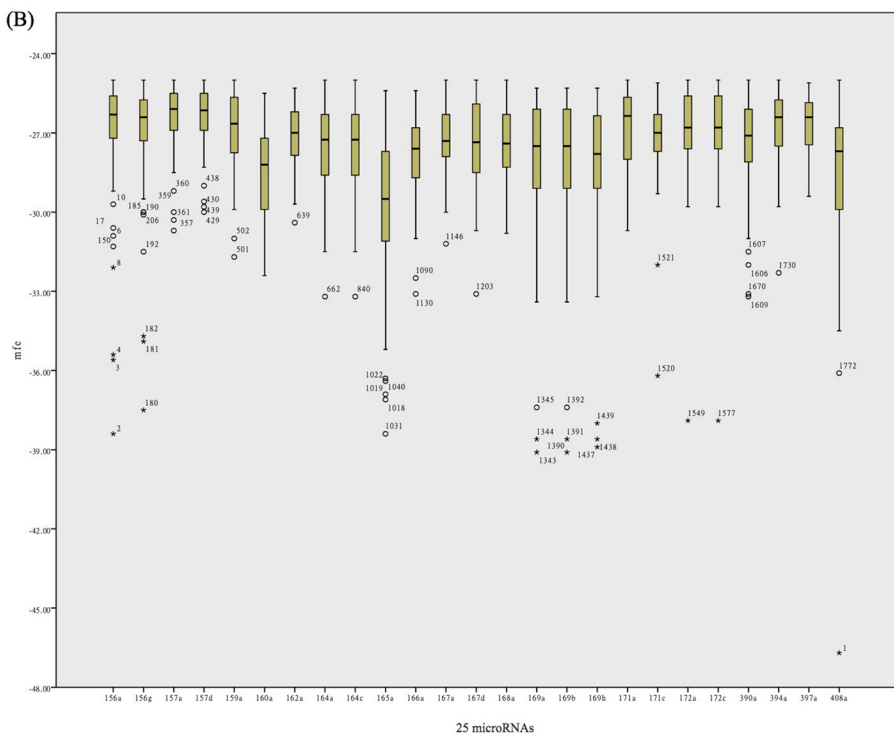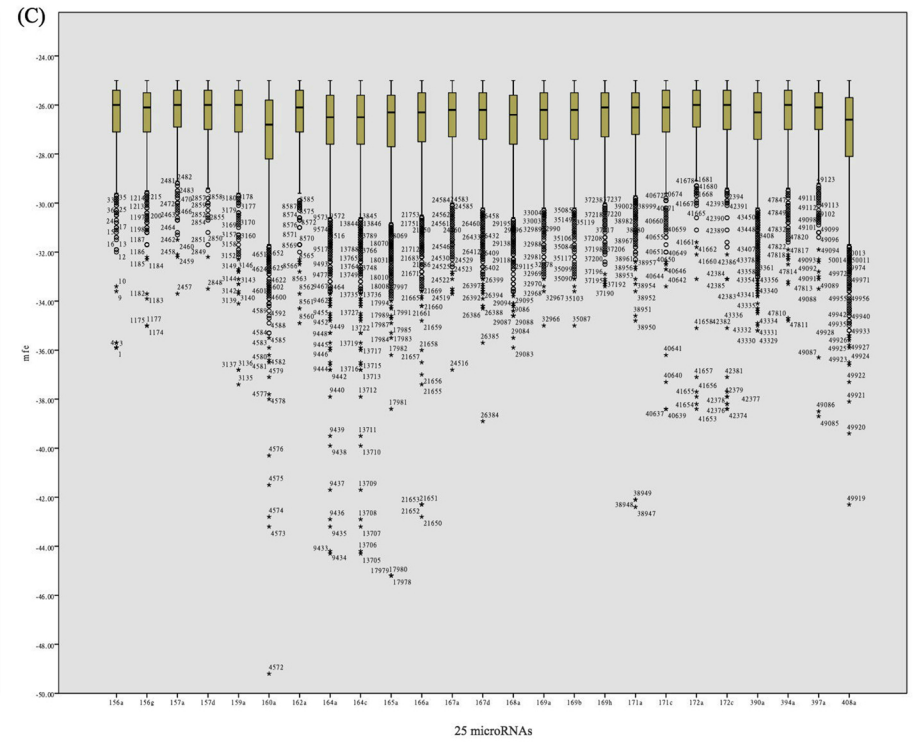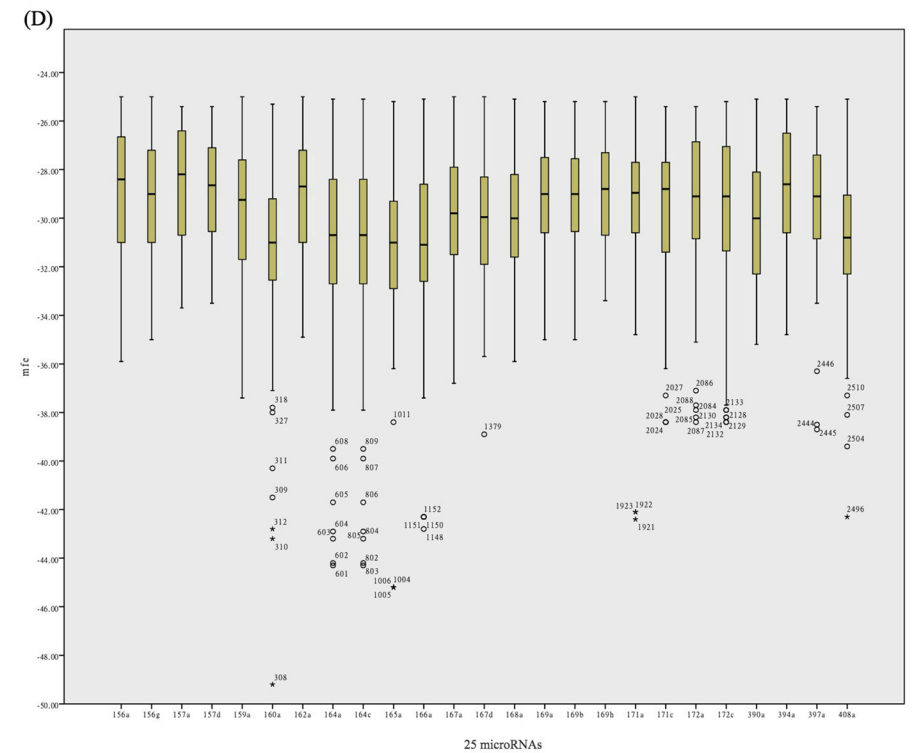

Supplement: Additional file 6: Figure S2. — Comparison between plant and human target distributions after the filtering process. (a) Original Arabidopsis targets. (b) Arabidopsis targets after screening. (c) Original Human targets. (d) Human targets after screening. There is a remarkable reduction of the noisy points between (a) and (b), and between (c) and (d), which strongly supports the effectiveness of our method and parameters, and provides a valid guide that can help explore the mechanism of cross-species miRNA targets. (PDF 8744 kb) [file 12918_2016_292_MOESM6_ESM.pdf]
